# C3 glomerulonephritis in multiple myeloma: A case report and literature review

**DOI:** 10.1097/MD.0000000000004843

**Published:** 2016-09-16

**Authors:** Guang Yin, Zhen Cheng, Cai-Hong Zeng, Zhi-Hong Liu

**Affiliations:** National Clinical Research Center of Kidney Diseases, Jinling Hospital, Nanjing University School of Medicine, Nanjing, China.

**Keywords:** C3 glomerulonephritis, C3 glomerulopathy, case report, complement 3, multiple myeloma

## Abstract

**Background:**

C3 glomerulonephritis (C3 GN) is a recently defined entity characterized by predominant C3 deposition in glomeruli due to abnormal activation of the alternative pathway of complement system. C3 GN has been reported to be associated with several systemic diseases. However, the association between C3 GN and multiple myeloma (MM) has not been well established.

**Methods:**

We herein describe a case presenting with C3 GN on top of MM.

**Results:**

A 64-year-old Chinese female presented with gross hematuria, renal dysfunction, anemia, and weight loss. Results of serum immunofixation assay and bone marrow biopsy confirmed the diagnosis of IgG-λ-type MM. In addition, renal biopsy demonstrated histological findings characteristic of C3 GN, including mesangial and endocapillary proliferation under light microscope, electron-dense deposits under electron microscope, and diffuse granular deposition of C3 with no immunoglobulin under immunofluorescence microscope. These histological findings, combined with low serum C3 level, suggested the occurrence of C3 GN in the context of MM.

**Conclusion:**

This case study provides additional evidence to the literature in terms of the association between C3 GN and MM. We hypothesize that C3 GN may present as a new variant of nephropathy in MM and the mechanism behind this association merits further study.

## 1 Introduction

C3 glomerulonephritis (C3 GN) is a recently defined glomerulonephritis characterized by glomerular deposition composed of C3 with minimal or no immunoglobulin.^[1,2]^ The disease is caused by abnormal activation of the alternative complement pathway and may be associated with several conditions that result in dysregulation of the pathway such as infection^[3]^ and monoclonal gammopathy.^[4]^ As a relatively newly recognized entity, data regarding the clinical and pathological features of C3 GN remain limited, and its association with other systemic/renal diseases is not well understood.

Multiple myeloma (MM) is a plasma cell neoplasm with a common feature of renal involvement. A variety of renal diseases have been observed in MM, of which myeloma cast nephropathy and monoclonal immunoglobulin deposition disease are the most frequent ones, followed by fibrillary glomerulonephritis, immunotactoid glomerulopathy, and crystalline histiocytosis as less frequent variants.^[5]^ To our knowledge, C3 GN has been described in a number of cases of MM in the literature,^[6–8]^ but the association between C3 GN and MM has not been well established. We herein describe a case presenting with typical features of C3 GN and MM to evidence the association of the 2 entities. In addition, we propose the hypothesis that C3 GN may be a new variant of nephropathy in MM.

## 2 Case report

A 64-year-old female presented to our hospital with a 3-month history of gross hematuria, proteinuria, renal dysfunction, anemia, and weight loss. Three months before presentation, she had an attack of gross hematuria with urinary urgency and dysuria, for which she started looking for medical attention at the local hospital. She was found to have gross hematuria (red blood cell count 80 × 10^6^/mL, pleomorphic type), elevated serum creatinine (2.9 mg/dL), anemia (hemoglobin 63 g/L), and hyperglobulinemia (41.0 g/L), and was treated with traditional Chinese medicine. However, she was not responsive to the treatment, and there was a rapid weight loss (10 kg) in the following 3 months. She was subsequently referred to our hospital for further management. The patient's medical and family history were otherwise unremarkable.

On admission, physical examination revealed blood pressure of 154/90 mm Hg, body mass index (BMI) of 22.0 kg/m^2^, and anemic palpebral conjunctiva. Urinalysis showed gross hematuria (red blood cell count 300 × 10^6^/mL, pleomorphic type), proteinuria (1.67 g/24 h), and elevated levels of *N*-acetyl-β-d-glucosaminidase (106.0 U/gCr, normal range ≤16.5 U/gCr) and retinol binding protein (18.0 mg/L, normal range ≤0.5 mg/L). Urinary output and osmolality was 1270 mL/24 h and 412 mOsm/kg·H_2_O, respectively.

Complete blood count showed severe anemia (hemoglobin 60 g/L) with normal counts of white blood cells and platelets. Kidney function was greatly reduced (serum creatinine 2.43 mg/dL, blood urea nitrogen 24.0 mg/dL, eGFR 20.5 mL/min/1.73 m^2^), and total serum protein was significantly increased (77.0 g/L) in the context of hypoalbuminemia (30.0 g/L) and hyperglobulinemia (47.0 g/L). Liver enzymes, serum electrolytes, and blood glucose were within normal range. Serum level of C3 was decreased (0.62 g/L, normal range 0.8–1.8 g/L), but levels of C4 and complement factor H (CFH) were normal. C3 nephritic factor (C3NeF) and antifactor H antibody were negative. Antinuclear antibody, antineutrophil cytoplasmic antibody, and antiglomerular basement membrane antibody were negative. Serology for hepatitis B was negative.

Given the presence of hyperglobulinemia, a quantitative study of immunoglobulin subclasses was performed, revealing a remarkable increase in immunoglobulin G (IgG, 41 g/L, normal range 7–16 g/L) and decrease in immunoglobulin A (IgA, <0.26 g/L, normal range 0.7–4.0 g/L) and immunoglobulin M (IgM, 0.18 g/L, normal range 0.4–2.3 g/L). Serum-free λ light chain level was greatly elevated (163.00 mg/L, normal range 6–20 mg/L), whereas free κ light chain level was normal. IgG-λ-type monoclonal immunoglobulin band was identified by immunofixation electrophoresis. Furthermore, a bone marrow biopsy showed hypercellularity with an increase in plasma cells (45.5%). Given these findings, a diagnosis of MM was made in spite of the absence of skull and pelvic bone lesions.

**Figure 1:**
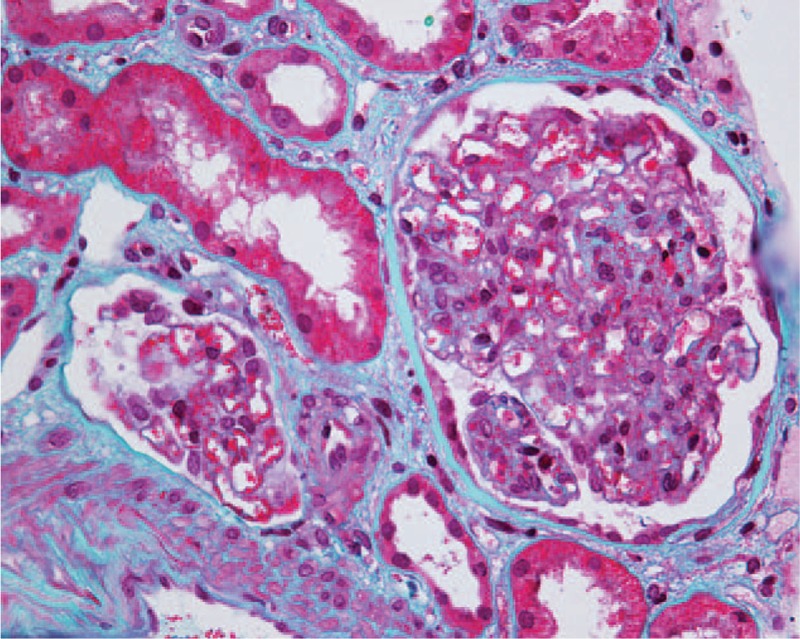
Light microscopy showing eosinophilic complexes in the mesangial and subendothelial areas. Masson trichrome stain, 400× magnification.

**Figure 2:**
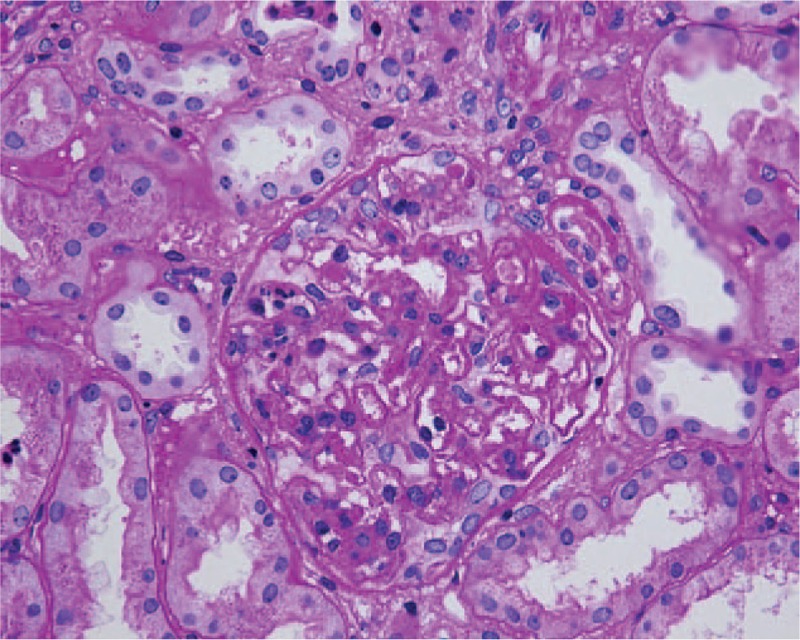
Light microscopy showing infiltration of mononuclear cells, plasma cells, and neutrophils in the interstitium. Periodic acid-Schiff stain, 400× magnification.

**Figure 3:**
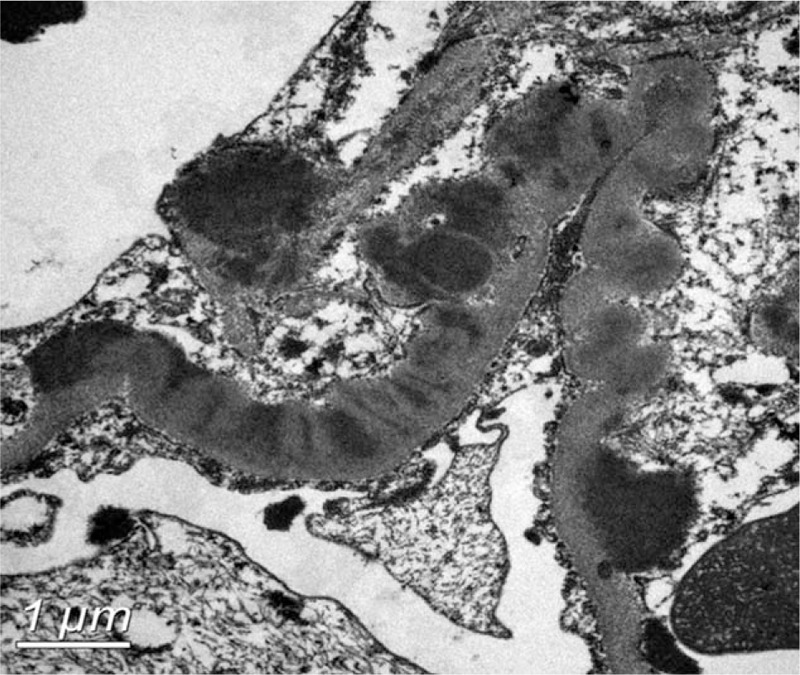
Electron microscopy showing electron-dense deposits in the subendothelial, intramembranous, and mesangial areas. Scale bar: 1 μm.

**Figure 4:**
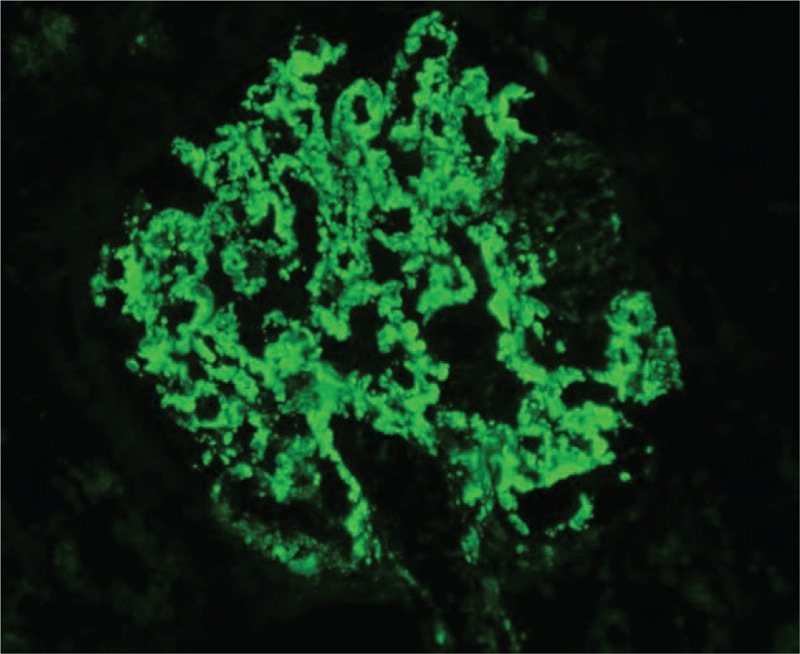
Immunofluorescence microscopy showing positive staining for C3 in the mesangium and along the capillary wall. 400× magnification.

The patient subsequently underwent a renal biopsy to evaluate renal damage. Histological findings were characteristic of C3 GN. Specifically, global mesangial matrix expansion and mesangial hypercellularity were noted under light microscope, with segmental thickening of Bowman's capsule, proliferation of endocapillary cells, and infiltration of mononuclear cells and neutrophils in some glomeruli. Eosinophilic complexes were observed in the subendothelial, intramembranous, and mesangial areas (Fig. 1). There were moderate tubulointerstitial lesions with a few mixed casts. Interstitial fibrosis was mild with moderate infiltration of monocytes, plasma cells, and neutrophils (Fig. 2). Congo red staining was negative. Additionally, electron microscopy identified dense deposits primarily in the subendothelial area, as well as in the mesangial, intramembranous, and subepithelial areas (Fig. 3). Segmental fusion of foot process was also noted. Immunofluorescence microscopy reveled positive staining for C3 in the mesangium and along the capillary wall (Fig. 4). Meanwhile, IgG, IgA, IgM, complement 1q (C1q), κ light chain, and λ light chain staining were negative.

Taken together, the patient was diagnosed with C3 GN and MM. She was treated with a chemotherapy regimen consisting of thalidomide and dexamethasone. Despite of a decrease in serum-free λ light chain level, there was no significant improvement in serum creatinine (3.16 mg/dL), hematuria (red blood cell count 2000 × 10^6^/mL), and anemia (hemoglobin 67 g/L) after 1 month. The patient is now in follow-up.

## 3 Discussion

In this study, we describe a rare case presenting with C3 GN on top of IgG-λ-type MM, further strengthening the recently recognized association between the 2 entities.

C3 GN has been reported to be associated with several systemic disorders, but its association with MM has not been well established. Development of C3 GN in the context of MM has been described in only 5 patients in the literature.^[6–8]^ The first case, characterized by MM and proliferative glomerulonephritis with granular glomerular deposition of C3, low serum C3, and positive C3NeF activity, was reported even before the term of C3 GN was introduced.^[6]^ The second case, who had smoldering MM and glomerulonephritis featured by isolated C3 deposits, was reported when the term of C3 GN had not been widely accepted.^[7]^ Recently, Cooper et al^[8]^ reported 5 cases with C3 GN and plasma cell dyscrasia, including 2 presenting with symptomatic myeloma and 1 with monoclonal gammopathy of renal significance that progressed to symptomatic myeloma, suggesting a potential relationship between C3 GN and MM. In the present study, we presented a case with typical clinicopathologic features of C3 GN and MM, providing additional evidence for the association between C3 GN and MM.

Based on the present study and previous reports, we hypothesize that C3 GN may be a new variant of renal manifestation in MM. However, this causal relationship needs further investigation. C3 GN is believed to be caused by dysregulation of the alternative pathway of complement system. This may be result from genetic mutations of proteins involved in the pathway such as CFH, complement factor I (CFI), and complement factor H related proteins (CFHRs).^[9]^ However, it is thought that genetic defect alone may not be sufficient to initiate the disease in many patients.^[2,4]^ A trigger factor that targets one of the components of the pathway is often required for the development of C3 GN, such as C3NeF, an autoimmune antibody against C3 convertase,^[10]^ and antifactor H antibody, which targets CFH. In this study, the negative results of C3NeF and antifactor H antibody excluded their roles in the pathogenesis of C3 GN in our patient. Instead, it is possible that the abnormal monoclonal immunoglobulin produced by MM acts as such a trigger factor that leads to abnormal activation of the alternative complement pathway and contributes to the development of C3 GN. The hypothesis that abnormal monoclonal immunoglobulin may cause kidney diseases by dysregulating the complement system has been proposed by Zand et al^[4]^ based on their observation in a case series of C3 GN associated with monoclonal gammopathy. Further studies are needed to verify this hypothesis and elucidate the effects of these abnormal immunoglobulins on the complement system.

The association between C3 GN and MM has a number of indications in clinical practice. First, as C3 GN is becoming better recognized among physicians, it warrants a search for underlying hematologic malignancy such as MM in patients with C3 GN. Second, it suggests the importance of treating underlying malignancy as part of the treatment of C3 GN. However, chemotherapy may not contribute to an improvement in renal function, depending on the stage of renal involvement.

In summary, this case study provides additional evidence for the association between C3 GN and MM. Further studies are needed to confirm this finding and clarify the mechanism behind the association.
